# Extent and mechanism of phase separation during the extrusion of calcium phosphate pastes

**DOI:** 10.1007/s10856-015-5615-z

**Published:** 2015-12-24

**Authors:** Rory O’Neill, Helen O. McCarthy, Eoin Cunningham, Edgar Montufar, Maria-Pau Ginebra, D. Ian Wilson, Alex Lennon, Nicholas Dunne

**Affiliations:** School of Mechanical and Aerospace Engineering, Queen’s University Belfast, Stranmillis Road, Belfast, BT9 5AH UK; Biomaterials, Biomechanics and Tissue Engineering Group, Department of Materials Science and Metallurgy, Technical University of Catalonia, BarcelonaTech (UPC), Av. Diagonal 647, 08028 Barcelona, Spain; Department of Chemical Engineering and Biotechnology, New Museums Site, University of Cambridge, Pembroke St, Cambridge, CB2 3RA UK; School of Pharmacy, Queen’s University Belfast, Lisburn Road, Belfast, BT9 7BL UK; School of Mechanical and Manufacturing Engineering, Dublin City University, Dublin 9 Glasnevin, Ireland

## Abstract

**Abstract:**

The aim of this study was to increase understanding of the mechanism and dominant drivers influencing phase separation during ram extrusion of calcium phosphate (CaP) paste for orthopaedic applications. The liquid content of extrudate was determined, and the flow of liquid and powder phases within the syringe barrel during extrusion were observed, subject to various extrusion parameters. Increasing the initial liquid-to-powder mass ratio, LPR, (0.4–0.45), plunger rate (5–20 mm/min), and tapering the barrel exit (45°–90°) significantly reduced the extent of phase separation. Phase separation values ranged from (6.22 ± 0.69 to 18.94 ± 0.69 %). However altering needle geometry had no significant effect on phase separation. From powder tracing and liquid content determination, static zones of powder and a non-uniform liquid distribution was observed within the barrel. Measurements of extrudate and paste LPR within the barrel indicated that extrudate LPR remained constant during extrusion, while LPR of paste within the barrel decreased steadily. These observations indicate the mechanism of phase separation was located within the syringe barrel. Therefore phase separation can be attributed to either; (1) the liquid being forced downstream by an increase in pore pressure as a result of powder consolidation due to the pressure exerted by the plunger or (2) the liquid being drawn from paste within the barrel, due to suction, driven by dilation of the solids matrix at the barrel exit. Differentiating between these two mechanisms is difficult; however results obtained suggest that suction is the dominant phase separation mechanism occurring during extrusion of CaP paste.

**Graphical Abstract:**

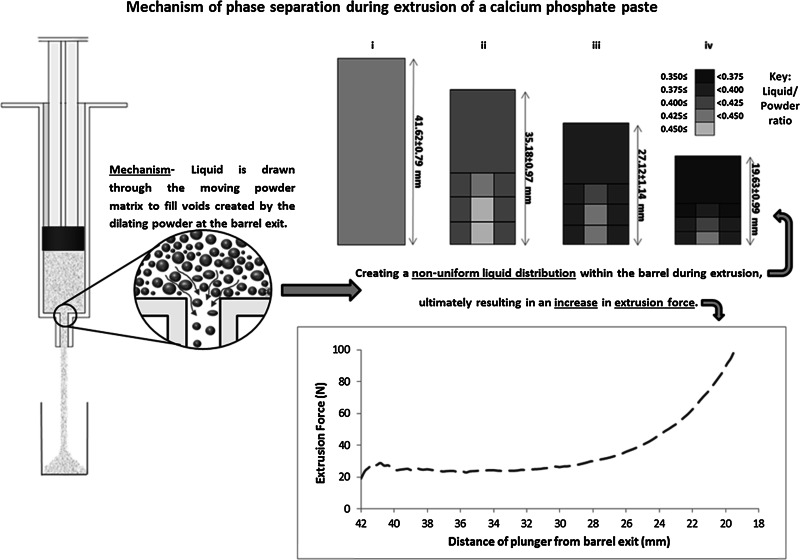

## Introduction

Since the discovery of calcium phosphate cement (CPC) by Brown and Chow in 1983 [1], it has seen clinical success in many dental and orthopaedic applications [2–5]. This is due to the potential for CPC based systems to mimic the natural mineral phase of bone and consequently be resorbed and replaced by host tissue [6, 7]. In addition, it has the ability to be moulded into bone defects and implant sites, then harden in situ to provide stability. However, in applications requiring injection or extrusion of CPC, separation of the powder (consisting of one or more compounds of calcium and phosphate salts) and liquid phase (water or an aqueous solution) is commonly observed. Phase separation results in extrudate with a significantly higher liquid content than the paste charged, or loaded, into the syringe [8]. A higher liquid content leads to a reduction in the mechanical properties of the hardened cement [9, 10] and a decrease in the viscosity of the unset CPC [11], increasing the risk of extravasation and embolism due to particle leaching [12], with potentially fatal consequences [13].

Currently ‘injectability’ is the most common method used to infer the extent of phase separation during extrusion of CPC. Injectability is usually defined as the ratio of mass of CPC able to be extruded until a maximum extrusion force is reached (taken as 100 N [14] to 300 N [15]) to the initial mass loaded into a syringe. Many studies have investigated injectability and various improvement methods have been established that include: (1) constituent modification—liquid to powder ratio (LPR) [14, 16], particle size [17–19] and shape [20], incorporating various additives [15, 21–24] and (2) altering external factors—such as plunger rate [8] and applying ultrasonic vibration [25]. Yet, it is widely accepted that at present no fully injectable CPC exists that meets the clinical requirements for minimally invasive surgical applications in the treatment of bone disease or trauma [26]. This is due to the fact that the application is highly constrained and methods to improve injectability can be detrimental to other crucial properties of the cement required for clinical success, namely sufficient cohesiveness and mechanical performance.

Optimisation of CPC injectability, within the constraints of surgical applications, is currently inhibited by the difficulty in determining the benefits of one improvement strategy versus another by cross-comparison of different studies. This is due to the lack of standard testing protocol; i.e. the syringe geometry, extrusion rate and force, LPR, particle size, and particle size distribution differ between studies. Furthermore, as injectability does not consider extrudate quality, improving injectability does not necessarily increase clinical suitability. Bohner and Baroud [27] highlighted this limitation, and proposed that injectability should be defined as the capacity of the CPC to remain homogeneous during the extrusion process. Consequently, studies have investigated paste homogeneity during extrusion of a calcium phosphate (CaP) based paste by measuring and comparing the LPR of extrudate and paste remaining in the syringe post-extrusion [8, 19]. Although it was confirmed phase separation was occurring, limiting injectability, the exact mechanism by which phase separation of CPC occurs is still unknown.

Phase separation is not specific to CPC systems and is known to occur during extrusion of biphasic pastes in the chemical, food, and pharmaceutical sectors. A number of studies have used both experimental and theoretical approaches [28–31], improving understanding of both phase separation mechanisms and methods to reduce phase separation. In short, the literature on phase separation during extrusion proposes three mechanisms:

(i) *filtration in the needle* (or die land), where the pressure exerted on the liquid phase causes it to flow along the die land more quickly than the solids, exacerbated with the formation of solid mats (regions of high SVF) [32];
(ii) *filtration* in the barrel, where the pressure exerted by the plunger on the paste causes the liquid phase to redistribute and the solids to undergo consolidation [33]; and
(iii) local *suction* at the barrel exit driven by the dilation of the solids matrix as it flows into the nozzle. If the solid volume fraction (SVF) of a paste is sufficiently high, particles must separate to enable flow, i.e. the powder must dilate. Liquid is then drawn through the powder network to fill voids created by the dilating powder [31, 34].

Although recent studies have increased understanding of the occurrence of phase separation during extrusion of CaP pastes [8, 19, 35], a detailed understanding of the mechanism and dominant drivers is still lacking. Appreciation of the dominant mechanism will increase knowledge of how parameters influence the extent of phase separation, and enable more efficient optimisation of injectable CaP materials to fully satisfy clinical requirements.

The aim of this study was to further improve understanding of phase separation and determine the dominant mechanism driving it; specifically, whether it is filtration in the needle, filtration in the barrel, or local suction at the barrel exit. More detailed insights, relative to previous investigations, were discussed via determination of the radial and vertical liquid distribution of paste located in the syringe throughout extrusion; and detailed analysis of extrusion parameters on paste flow and phase separation characteristics relative to established extrusion and phase separation mechanisms observed in other paste based systems.

## Materials and methods

These experiments were designed to ensure adequate measurement of the extent of phase separation throughout extrusion of a CaP paste, over various extrusion parameters. Specific parameters investigated were: (1) injectability, (2) extrusion force, and (3) liquid content of the extrudate and paste located within the barrel throughout extrusion. The flow of the powder and liquid components within the barrel during and post-extrusion were examined using powder tracing tests. These tests specifically observed the displacement of powder (i.e. powder flow) and were determined using paste produced from dyed CaP powder of differing colours, which created a ‘striped effect’ when loaded into the syringe. The flow of liquid was estimated by determination of the radial and vertical liquid distribution within the barrel during and post-extrusion. Further information on the phase separation mechanism was generated by altering the syringe geometry in the form of the taper at the barrel exit.

### Powder characterisation

The powder component was a commercial grade of β-tri-calcium phosphate, β-TCP, Ca_3_(PO_4_)_2_ (Sigma-Aldrich, UK). The particle size distribution of the β-TCP powder was analysed by laser diffraction (LS 13 320 Beckman Coulter, UK). The specific surface area (SSA) was determined by nitrogen adsorption according to the Brunnauer–Emmet–Teller (BET) method (ASAP 2020 Micromeritics, UK). The plastic limit (PL) was determine using a similar method to Bohner and Baroud [27]. The LPR at which the powder formed a ‘pasty block’ was determined by the drop-wise addition of distilled water (dH_2_O) to 3 ± 0.1 g of powder. The packing behaviour of the powder, maximum solid volume fraction (SVF_max_) reached under compaction, was determined using a Rigden voidage device according to EN 1097-4. Helium pycnometry was performed to obtain the specific density of the β-TCP powder (AccuPyc 1330 Micromeritics, UK), for use in the SVF calculations.

### Paste preparation

To produce CaP paste, the powder and liquid (dH_2_O) components were mixed manually, at initial LPR values of 0.4, 0.425 and 0.45 g/g, using a bowl and spatula arrangement for 45 s until a homogeneous paste resulted. These initial LPRs were chosen to facilitate detailed examination of the phase separation mechanism. Pastes of β-TCP and dH_2_O have previously been selected as model systems by several groups when investigating CPC [8, 18, 36], as they have similar properties to CPC. Moreover the CaP paste has longer setting time [18] and is less expensive.

### Extrusion of paste

**Fig. 1:**
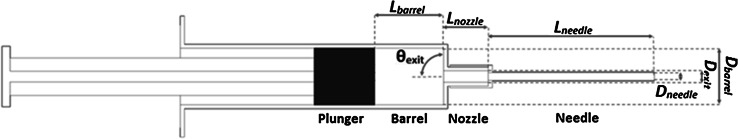
Syringe geometry and associated nomenclature (not to scale)

Pastes were carefully loaded into a 10 mL Luer lock MiniMix™ syringe (Summit Medical Ltd., UK). The dimensions of the MiniMix™ syringe were as follows: diameter of barrel, D_barrel_ = 13.5 ± 0.01 mm, diameter of exit, D_exit_ = 3 ± 0.01 mm, angle of barrel exit, θ_exit_ = 90 ± 0.1° and length of nozzle L_nozzle_ = 12 ± 0.01 mm (Fig. 1). Using an EZ50 Universal Materials Testing Machine (Lloyd Instruments Ltd., UK), paste was extruded at a constant volumetric flow rate until a maximum force (100 N) was reached. The force required to extrude the paste was recorded using the NEXYGEN 4.1 software (Lloyd Instruments Ltd., UK) and was presented (e.g. Fig. 3) alongside the mean extrusion pressure, which was calculated from extrusion pressure = 4 × extrusion force$/\pi {\text{D}}_{\text{barrel}}^{2}$.

Three plunger rates were investigated: 5, 10 and 20 mm/min, corresponding to flow rates of 0.012–0.048 mL/s. These flow rates were selected to: (1) ensure phase separation occurred, (2) allow adequate time for accurate extrudate sampling and (3) include flow rates representative of injection rates observed during vertebroplasty procedures [37]. To investigate the effect of needle geometry, various needle lengths (L_needle_ = 35, 70 and 105 mm) and diameters (D_needle_ = 1.6, 1.8 and 2.16 mm) were used, representative of diameters used in percutaneous vertebroplasty procedures [38]. To investigate the effect of θ_exit_, conical inserts were manufactured in-house to fit in the syringe barrel, creating a conical taper from D_barrel_ to D_exit_; values of θ_exit_ investigated were 90° (square ended, no insert), 55 ± 0.1° and 45 ± 0.1°.

### Extrusion analysis

#### Injectability

The mass of extrudate, *M*_*extrudate*_ and paste remaining in the barrel, *M*_*plug*_, was determined post extrusion. Injectability was defined as the percentage of *M*_*extrudate*_ relative to the sum of *M*_*extrudate*_ and *M*_*plug*_ until a maximum load of 100 N was achieved (Eq. 1) [27].

1 $$Injectabilty (\%) = \frac{M_{extrudate}}{{M_{extrudate}} + {M_{plug}}} \times 100$$

#### Phase separation and liquid migration

**Fig. 2:**
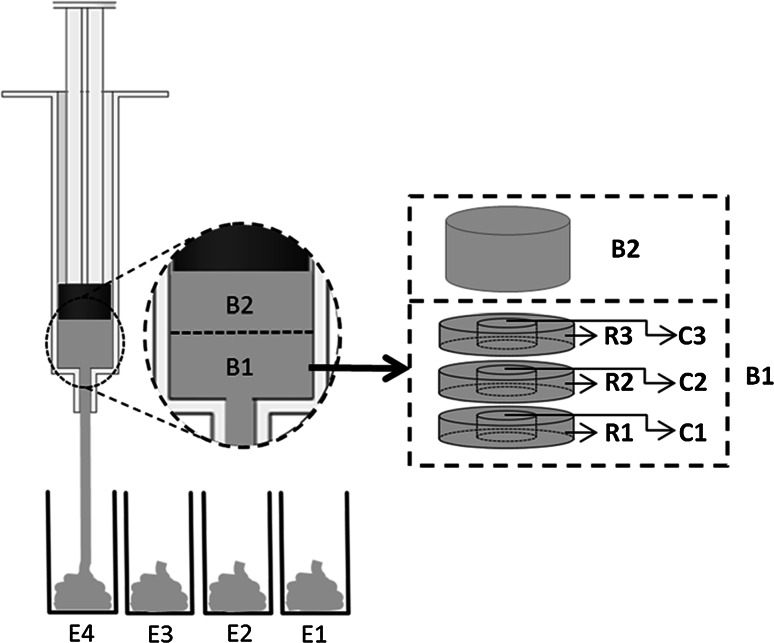
Schematic showing collection of samples of extrudate (E1–E4), the paste remaining in the syringe barrel (B1 and B2) and division of element B1 into rings (R1–R3) and cores (C1–C3) post extrusion

The extrudate exiting the syringe was collected as four consecutive samples of approximately equal volume, labelled (E1–E4), Fig. 2. The required sampling volume was obtained by running a preliminary extrusion test, determining the volume of extrudate post-extrusion, and dividing by four. The paste remaining in the barrel post-extrusion was divided into two equal parts (labelled B1 and B2). Each sample was initially weighed and dried in an oven at 100 °C until a constant weight was reached. Pre-drying and post-drying weights were used to determine LPR using a protocol similar to that reported by Habib et al. [8]. The LPR of extrudate samples and of paste remaining in the barrel was determined and the extent of phase separation, defined as the percentage increase in LPR of extrudate relative to initial LPR, calculated.

In order to determine the liquid distribution at the barrel exit, the same process was conducted, however when B1 was removed, it was divided into two or three equal slices depending on CaP paste quantity. The core region (diameter 6 mm) was then removed from each slice, and the LPR of each slice and the core was measured (Fig. 2). The liquid distribution within B2 was not investigated as preliminary results showed uniform LPR within this region.

#### Solids flow regime

To enable visualisation of the flow patterns for the solids, a dyed powder was used. The β-TCP powder was dyed using water-insoluble Pantone coloured ink (Esselte Ltd, UK), added as one drop of ink per gram of powder. The powder was manually mixed until a uniform colour was reached. The dyed powders were dried in an oven held at 37.5 ± 0.1 °C for 12 ± 0.1 h and then stored in a desiccator until ready for use. The different coloured CaP pastes were mixed individually with the same LPR and then loaded into the syringe in sequence to create a striped pattern. Following extrusion, the paste remaining in the syringe was carefully removed and sliced along a diametrical plane direction, using a blade and a purpose made holder. This revealed the flow pattern at the nozzle in the longitudinal direction.

### Statistical analysis

Each test was repeated a minimum of three and up to a maximum of six times. Where feasible, results were analysed for statistical significance. For injectability results, one-way or two-way analysis of variance (ANOVA) was conducted, following confirmation of normality of data and homogeneity of variance. If significant, a Tukey’s Test was then used to conduct a comparison of means. Where data from two independent samples were required to be tested for statistical significance a two-tailed *t* test was used. For all other dependents, regression analysis was used. A probability value of *P* ≤ 0.05 was considered significant. All statistical analysis was performed using SPSS Statistics 20.0 (IBM Corporation, USA).

## Results

### Powder characterisation

The β-TCP powder had a median particle size (D_50_) of 2.3 µm and polydispersity (d_span_) of 3.9 ± 0.8. The SSA obtained was 2.10 ± 0.05 m^2^/g (Table 1). Using the specific density value obtained (3.047 ± 0.001 g/cm^3^) SVF_Rigden_ was calculated as 0.535 ± 0.014. The PL was observed at an LPR of 0.356 ± 0.007 (SVF = 0.480 ± 0.005).

**Table 1:** Particle size distribution parameters, surface area and packing ability estimates

	Particle size (microns)				SSA (m^2^/g)	Specific density (g/cm^3^)	PL (LPR)	SVF_max_
	d_10_	d_50_	d_90_	d_span_				
Mean	0.5	2.3	9.6	3.9	2.10	3.047	0.356	0.535
SD	0.2	0.0	2.2	0.8	0.05	0.001	0.007	0.014

Particle size values presented to 1 decimal place due to resolution of analyser (analysis range 0.017–2000 µm)

### Plunger rate and initial LPR

**Fig. 3:**
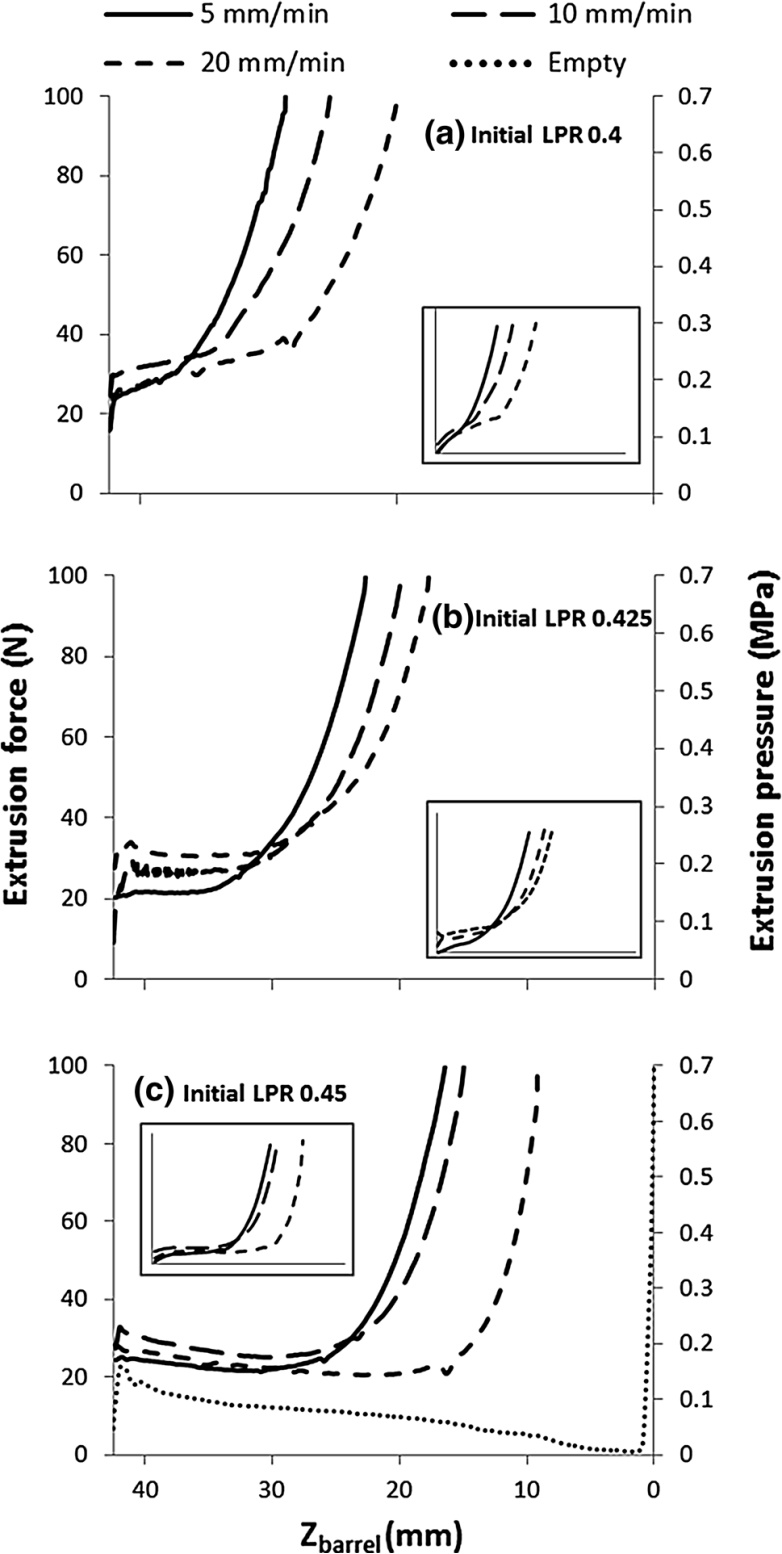
Effect of plunger rate on extrusion force-plunger displacement profiles for pastes with initial LPR of: **a** 0.4, **b** 0.425 and **c** 0.45. Z_barrel_ is the distance of the plunger from the barrel exit. *Square* entry (*θ* = 90°). *Dotted line* in **c** represents force required to displace plunger without paste in the barrel, i.e. the force required to overcome plunger friction. *Inset* shape of the extrusion profiles with plunger friction removed

The extrusion pressure showed an initial steady pressure, or slight elevation, that increased rapidly nearing the end of extrusion (Fig. 3). Present in the extrusion profile for an empty syringe (Fig. 3c), was a steady decrease in extrusion pressure, indicating that the barrel diameter changed steadily along its length, i.e. it has a slight taper. The contribution of the paste extrusion to the pressure was estimated from the difference between the filled and empty profiles, examples are presented as an inset in Fig. 3a–c.

**Fig. 4:**
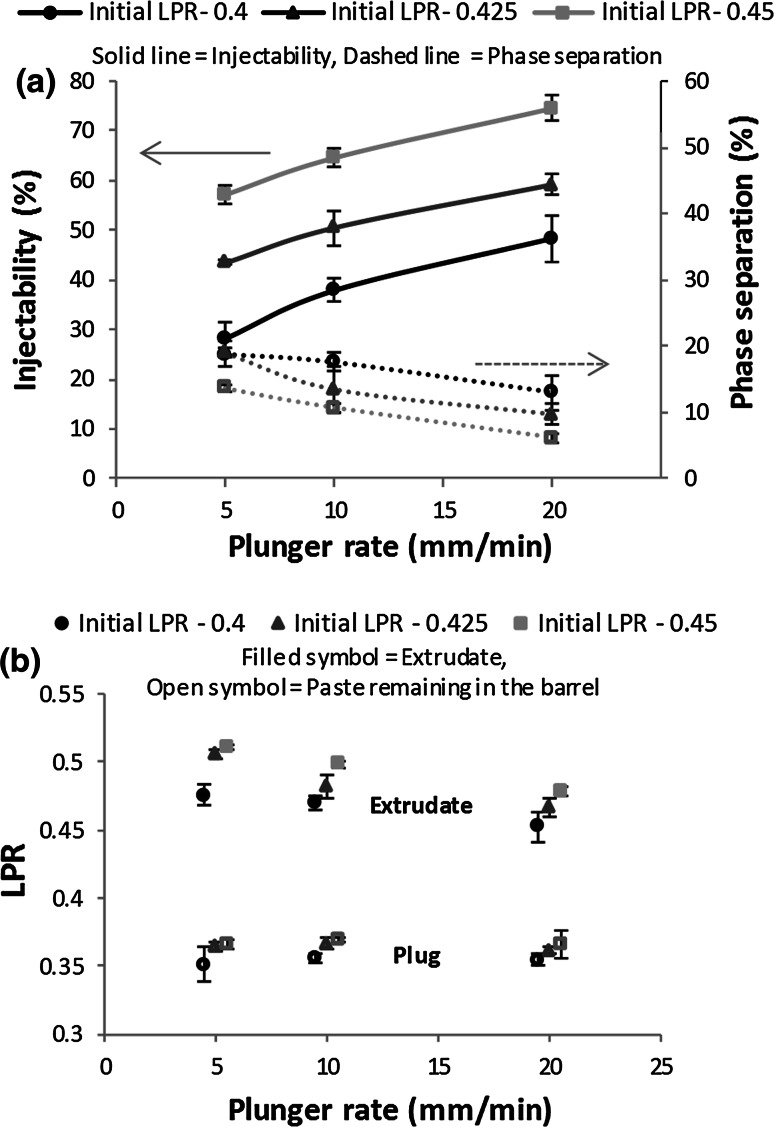
Effect of plunger rate on: **a** injectability and extent of phase separation, and **b** LPR of extrudate and paste remaining in barrel (plug) following extrusion of 6 mL of paste at various initial LPR and plunger rates. *Square* entry (*θ*_exit_ = 90°). *Error bars* represent SD

Increasing initial LPR and plunger rate significantly increased injectability of these CaP pastes (*P* < 0.001), Fig. 4a. The average LPR of the extrudate was consistently higher than that of the initial paste, Fig. 4b. Extrudate LPR was reduced by a decrease in initial LPR and an increase in plunger rate (*P* < 0.001 and *P* < 0.001, respectively, R^2^ = 0.852). The LPR of paste recovered from the barrel (labelled ‘plug’) was considerably lower than the initial LPR. No significant effect with regards to plunger rate on plug LPR was noted, however a significant effect was observed for initial LPR on plug LPR (*P* = 0.789, *P* < 0.001, respectively R^2^ = 0.391).

**Fig. 5:**
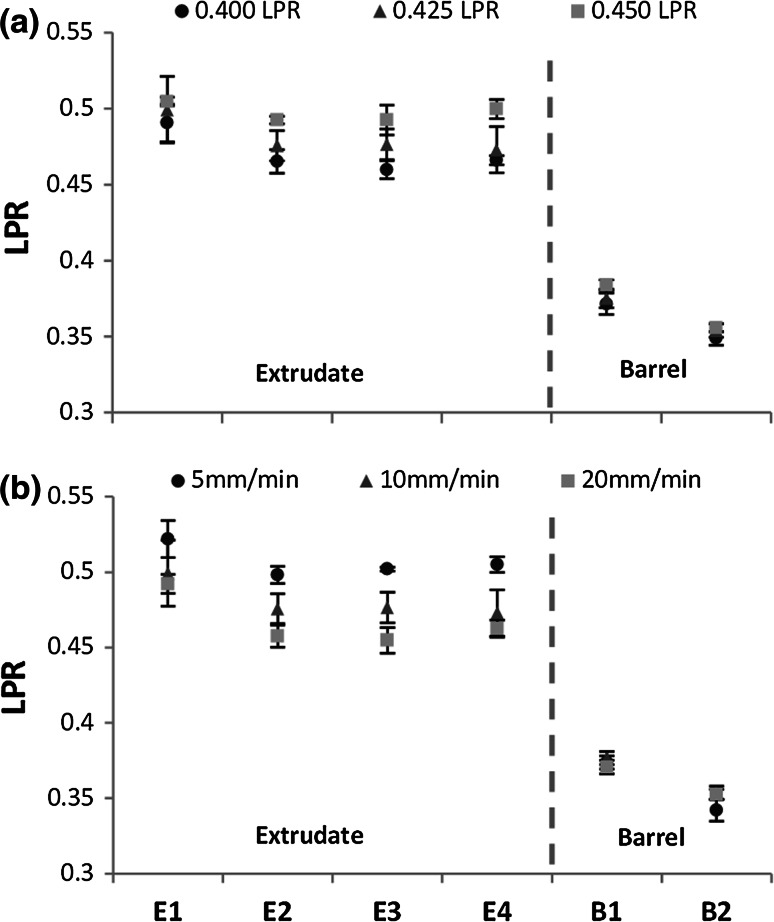
LPR distribution for consecutively extruded samples (E1–E4) and the paste remaining in the barrel (B1 exit side; B2 plunger side, see Fig. 2) for **a** different initial LPR values, plunger rate 10 mm/min; **b** different plunger rates, initial LPR of 0.425. *Square* entry (*θ*_exit_  = 90°). *Error bars* represent the standard deviation

The local LPR values (Fig. 5) show that the extrudate composition was relatively constant, although the initial sample (E1) had a slightly higher liquid content. All extrudate samples had a higher LPR than the initial paste, this varied slightly with initial LPR (Fig. 5a) and plunger velocity (Fig. 5b). The paste remaining in the barrel post-extrusion was drier at the plunger side than the paste at the barrel exit, the LPR of paste at the barrel exit being 7.00 ± 1.98 % higher than the paste at the plunger side (Fig. 5a, b).

### Geometry of delivery device

**Fig. 6:**
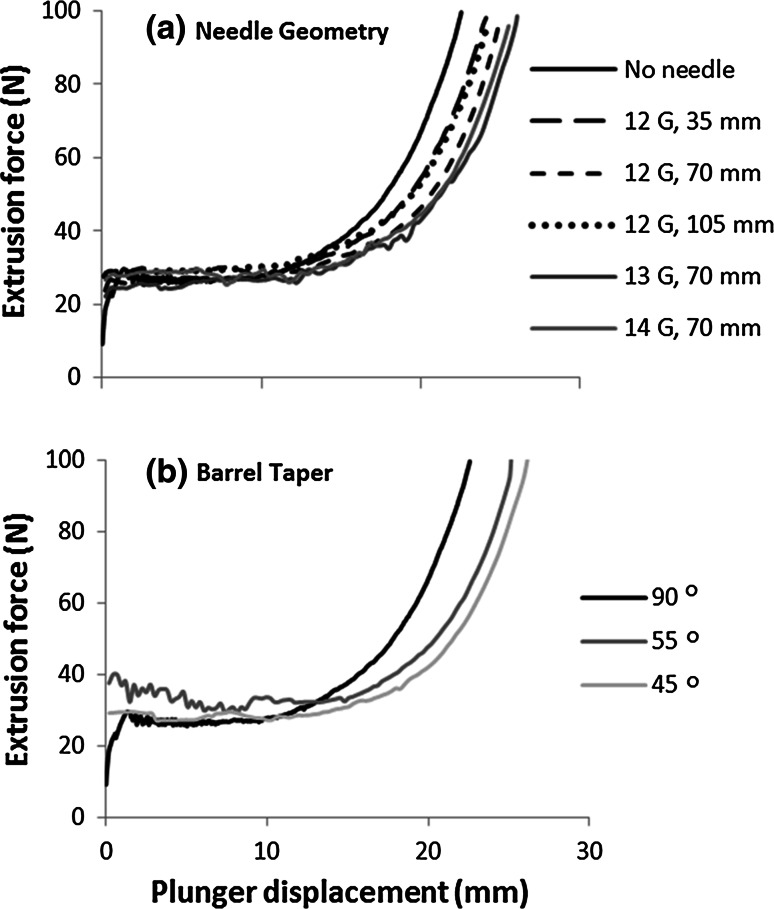
Effect of **a** needle geometry and **b** barrel taper on extrusion force-plunger displacement profiles for pastes with initial LPR of 0.425

Needle diameter and length had no significant effect on injectability (*P* = 0.628 , *P* = 0.920), on extrudate LPR (*P* = 0.106, *P* = 0.527, R^2^ = 0.229), or the LPR of the plug (*P* = 0.097, *P* = 0.167, R^2^ = 0.318), Table 2. However, compared to extrusion without a needle, addition of a needle slightly improved injectability (4.8 % increase, *P* = 0.002) but had no significant effect on LPR of extrudate (*P* = 0.326) or plug (*P* = 0.690). Additionally, needle geometry did not appear to influence extrusion pressure, Fig. 6a.

**Table 2:** Effect of needle dimensions on extrusion of CaP paste (6 mL)

	Needle diameter (mm)/gauge (G)				
	2.16/12			1.80/13	1.60/14
	Needle length (mm)				
	35	70	105	70	
Injectability (%) (mean ± SD)	54.871 ± 3.628	54.528 ± 0.899	54.056 ± 1.943	56.208 ± 1.690	56.267 ± 3.058
LPR of extrudate (mean ± SD)	0.471 ± 0.005	0.474 ± 0.005	0.475 ± 0.013	0.486 ± 0.012	0.482 ± 0.007
LPR of barrel (mean ± SD)	0.363 ± 0.005	0.367 ± 0.001	0.367 ± 0.002	0.369 ± 0.005	0.370 ± 0.006

Initial LPR = 0.425; plunger rate 10 mm/min; D_barrel_ = 13.5 mm, D_exit_ = 2.3 mm

 Tapered nozzle entries of 55° and 45° significantly improved injectability (*P* = 0.002 and 0.001, respectively) over the square ended die, but no significant difference was observed between injectability for the two taper angles tested, *P* = 0.968 (Table 3). The mean extrudate LPR decreased as the taper angle decreased (*P* < 0.001, R^2^ = 0.699) but phase separation was still observed: the LPR of the paste remaining in the barrel was considerably lower than the initial LPR. The extrusion force profiles of the tapered nozzle entries are similar in shape to the square entry die, i.e. an initial steady force followed by an abrupt increase (Fig 6b).

**Table 3:** Influence of nozzle entry geometry on injectability and liquid content of extrudate and paste remaining in the barrel

Angle of taper (°)	90	55	45
		
Injectability (%) (mean ± SD)	50.454 ± 3.890	62.841 ± 3.056	63.567 ± 1.985
LPR of extrudate (mean ± SD)	0.482 ± 0.010	0.449 ± 0.025	0.434 ± 0.012
LPR in the barrel (mean ± SD)	0.366 ± 0.005	0.361 ± 0.011	0.368 ± 0.017

Initial LPR = 0.425; plunger rate 10 mm/min

There was no significant difference between the LPR values of the paste remaining in the barrel for the various θ_exit_ values investigated (*P* = 0.915, R^2^ = 0.001). In fact, the LPR of paste remaining in the barrel post-extrusion remained similar throughout the study (0.364 ± 0.007). This indicates that 0.364 is the minimum LPR, or LPR_min_ (SVF = 0.474), that can be achieved under 100 N (pressure of 0.7 MPa) with the syringe geometry used in this study.

### Flow regime of liquid and powder components during extrusion

**Fig. 7:**
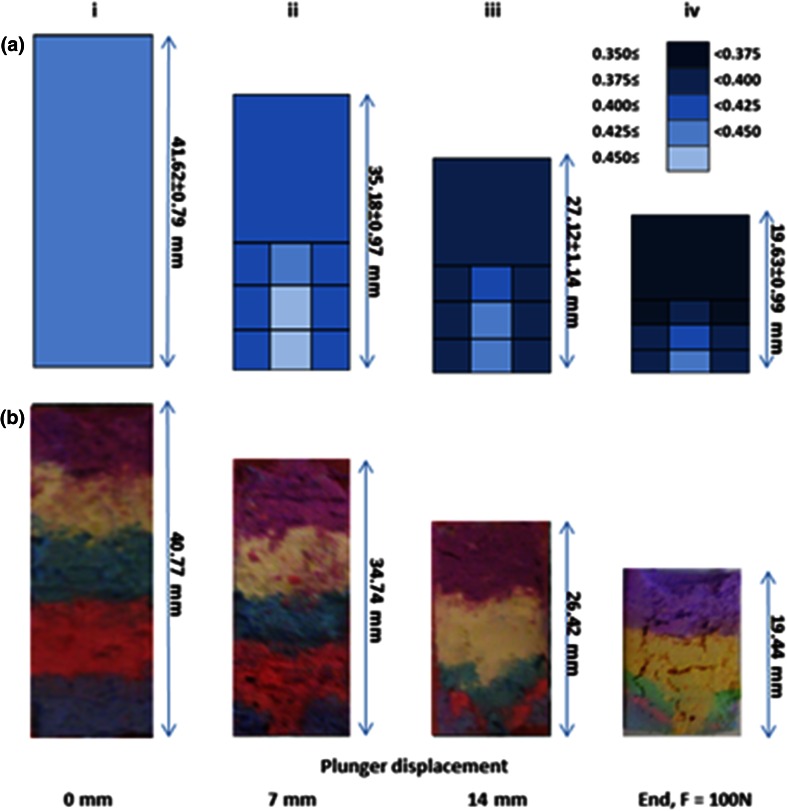
Distribution of paste liquid content (series **a**) and powder history (series **b**) for extrusion of paste with initial LPR of 0.425 through a square entry die (θ_exit_ = 90°) at plunger rate of 10 mm/min. Series **a** legend shows LPR colour coding. Series **b** (0 mm) shows paste initially loaded in bands of different colours (Color figure online)

**Fig. 8:**
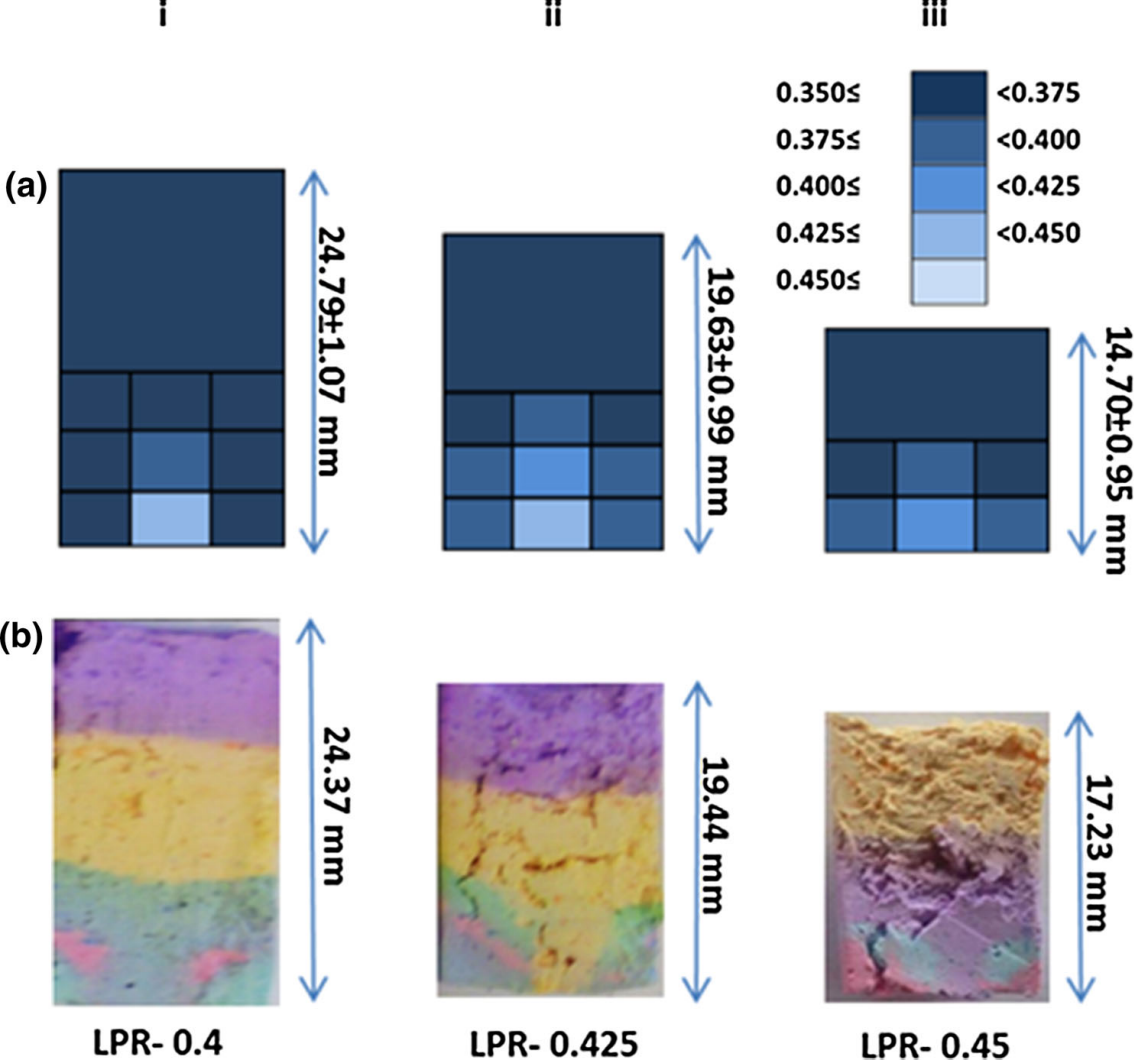
Distribution of paste liquid content (series **a**) and powder history (series **b**) post-extrusion of CaP pastes with initial LPR of 0.4, 0.425 and 0.45 through a *square* entry die (*θ*_exit_ = 90°) at plunger rate of 10 mm/min. Series **a** legend shows LPR colour coding (Color figure online)

Liquid content distributions in Fig. 7a show that, during extrusion, the LPR of paste in the barrel decreased, except for the material in the paste core regions (C1 and C2). The LPR in the latter remained similar to that of the extrudate. The same trends were observed for all initial LPRs investigated (Fig. 8a): the material in the core region had a higher liquid content while the LPR in the adjoining regions (R1–R3) were close to the rest of the paste remaining in the barrel (B2). The dyed paste studies (Figs. 7b, 8b) showed that the initially striped layers changed shape on approach to the nozzle entry. Zones of stagnant material were generated at the corners with the square-ended geometry for all LPR values. These stagnant zones are also regions of lower liquid content (R1–R3) and require high extrusion forces to deform and flow.

**Fig. 9:**
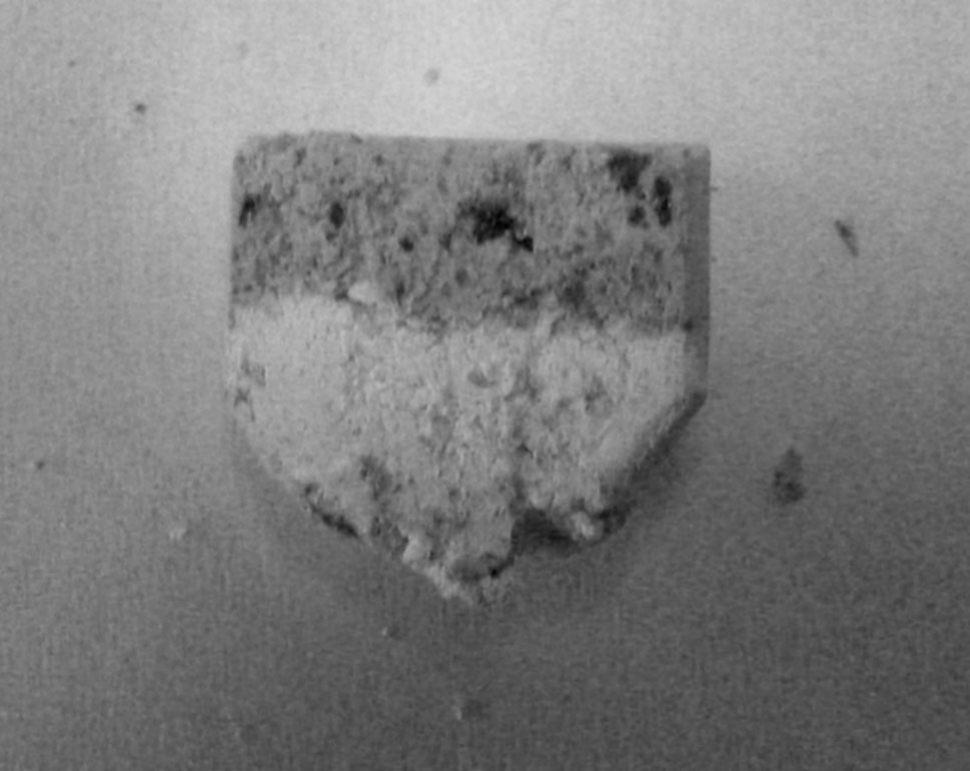
Cross-section of powder distribution following extrusion of paste with initial LPR = 0.425 through tapered barrel exit, *θ*_exit_ = 45°, at a plunger rate of 10 mm/min

In contrast, the cross-section of powder distribution following extrusion of paste with initial LPR = 0.425 through a tapered barrel exit, θ_exit_ = 45°, at a plunger rate of 10 mm/min showed no evidence of a static zone (Fig. 9).

## Discussion

In the present study the liquid content of extrudate, liquid distribution and flow of powder within the syringe barrel were measured throughout extrusion to better understand the mechanism and drivers of phase separation. Measurements made over several extrusion parameters and paste compositions enabled the characteristics of phase separation during extrusion of CaP pastes to be compared to established phase separation mechanisms observed during the extrusion of other biphasic pastes. The main trends observed and relationships between the measured parameters and phase separation are first discussed. Following this, the relationship between the findings and the three phase separation mechanisms discussed earlier (filtration in the needle, filtration in the barrel, and local suction at the barrel exit) are used to propose a dominant phase separation mechanism for non-reactive CaP pastes.

### Initial LPR and plunger rate

Increasing LPR was found to increase injectability (Figs. 3, 4), in agreement with previous studies [14, 16]. However, this was somewhat of an artificial increase. Higher initial LPR, resulted in a larger quantity of liquid to be redistributed until LPR_min_ was reached; this highlights the limitation of injectability as a measure of extrusion efficacy. Increasing plunger rate reduced phase separation. Plunger rate has been seen to be a significant influencing factor in phase separation of CaP [8] and other pastes [39, 40]. The increase can be attributed to less time available for the liquid to migrate or redistribute due to a reduction in residence time of the paste within the barrel [28]. This results in lower extrudate LPR at higher plunger rates. Therefore, increasing plunger rate decreases liquid supply to the extrudate from the paste in the barrel, resulting in a larger quantity of paste able to be extruded until LPR_min_ was reached and extrusion ceased (Fig. 3).

Both LPR and plunger rate were observed to have an effect on quantity and quality of extrudate. Interestingly, although there was variation in final plunger displacement, the extrusion profiles, were very similar in shape, initially exhibiting a slight gradient (Fig. 3a, b) or plateau (Fig. 3c) followed by an abrupt rise. Comparable extrusion profiles were observed in other CaP pastes extrusion studies [18, 19, 41]. It has been postulated that in the period before the abrupt increase in force, no phase separation takes place [41]. However, in this study it was evident that phase separation occurred throughout the extrusion process, as the extrudate LPR remained relatively constant (Fig. 5).

The initial extrusion force was relatively consistent at 25 N (0.2 MPa) for all pastes and does not show a systematic dependency on paste composition or plunger rate. Inspection of the empty test profiles suggests that the plunger seal friction provides the major contribution to the initial force. Therefore, as the initial force required to overcome plunger friction appears to be larger than the force required to initiate paste extrusion, the variation associated with the initial extrusion force will be dominated by variation in plunger friction. As a result, information regarding the initial extrusion force of the paste, and the investigated variables’ effect on it, is likely to be lost, highlighting a limitation of this study.

### Geometry of delivery device

The geometry of the device investigated in this study can be divided into two categories: (1) pre- and (2) post-barrel exit. Altering the angle of the barrel exit, affecting flow pre-barrel exit, significantly reduced extent of phase separation and increased injectability. The needle gauge or length, which determines the paste flow after it exits the barrel, had no significant effect on the extrudate quantity or quality (Table 2). It was found that needle addition slightly improved injectability (0.48 %) but did not significantly alter phase separation compared to extrusion with a syringe alone (90° in Table 3). In a study by Habib et al. [8], it was observed that addition of a needle slightly decreased injectability (2.5 %), whereas Montufar et al. [17] reported no significant difference between the extrusion of non-reactive CaP pastes with or without a needle. However, Montufar et al. did find needle geometry had an effect on the injectability of CPC. Burguera et al. [16] and Fatimi et al. [42] also found needle geometry had a significant effect on the extrusion of CPC, however both studies used a more viscous liquid phase (hydroxypropyl methylcellulose-water solution) and no phase separation was exhibited.

In these experiments, the insensitivity of the extrusion pressure (Fig. 6a), injectability and phase separation (Table 2) to needle geometry indicate that the contribution of the needle geometry to phase separation was negligible. The use of a tapered barrel exit improved injectability noticeably (Table 3). This is a significant finding, as this improvement method does not require altering composition of CPC. It has been suggested that tapered barrels will reduce extrusion pressure [43, 44], which in part could explain the reduction of phase separation when an acute θ_exit_ was used. However, due to the similarities of the pressure displacement profiles, this appears not to be a contributing factor in this study (Fig. 6b). The improvement has been attributed to manipulation of the flow pattern of the paste at the barrel exit reducing static zone formation.

### Flow regime of liquid and powder parts within syringe barrel during extrusion

Static zones, located at the corners of the square ended dies, and an inhomogeneous liquid distribution were evident from powder tracing and liquid distribution tests. A non-uniform liquid distribution was evident in Fig. 5, with the paste at the barrel exit being higher in water content than the paste located at the plunger side. A similar trend was observed by Habib et al. [8]. However this study has gone further to determine the radial distribution of the liquid, throughout the extrusion process. It was found during extrusion that paste located at the barrel wall and plunger side steadily dewatered throughout extrusion, increasing yield strength. The liquid migrated towards the core of the syringe. This inhomogeneous liquid distribution had a significant effect on the extrusion process. The lower liquid content in regions R1 and R2 (Fig. 7) was associated with formation of static zones. As the powder matrix within this zone was stationary the paste readily released excess liquid, increasing in yield stress, and also contributing to a higher LPR extrudate. Static zones have been observed in the extrusion of several pastes, indicating wall friction and yield stress behaviour within the paste system [28, 45, 46]. The shape of these zones are dependent on the competition between the yield stress of the material and the friction condition at the zone’s boundary [46]. In this study, size of the static zones increased as extrusion (and phase separation) continued, evident in the powder tracing results (Fig. 7b). Within and above these zones, the paste steadily dewatered growing stiffer. The paste at the core, C1, maintained a relatively constant LPR throughout extrusion, Figs. 7 and 8. Therefore it appeared paste adjacent to the lateral wall of the barrel resisted the pressure exerted by the plunger, irrespective of the LPR in the core, until the maximum plunger force was reached.

Similar trends were observed for all parameter combinations investigated; i.e. static zones and similar LPR distributions (Fig. 8a, b). No static zones were evident when tapered barrel exits were used. The absence of static zones (Fig. 9) with tapered exits means that powder was not lost to the formation of these features, as observed in extrusion with square ended dies—therefore injectability increased.

### Mechanism of phase separation

In these experiments, the insensitivity of the extrusion force and phase separation to needle geometry indicates that mechanism (i), filtration along the needle, was not active in these cases, (Fig. 4). If mechanism (i) was active the addition of a needle would be expected to increase phase separation and alter the pressure profile. It is apparent phase separation occurred within the barrel and the liquid flowed toward the barrel exit at a faster rate than the powder. This caused the paste within the barrel to become stiffer, increasing extrusion pressure. The flow rate of liquid will be dependent on the pore pressure (pressure exerted on the liquid) and permeability of the powder matrix.

What is not clear is if (1) the liquid was forced downstream by an increase in pore pressure as a result of powder consolidation due to the pressure exerted by the plunger (mechanism (ii)) or (2) the liquid was drawn from paste upstream, towards the exit due to local suction, driven by dilation of the solids matrix at the barrel exit (mechanism (iii)). It may be the case that both mechanisms contribute to phase separation.

In this study, the extrudate LPR remained relatively constant throughout extrusion for all parameters tested. The reasons behind the constant extrudate LPR and the influence the imposed conditions had on the extrudate LPR reached is not fully understood, but the results suggest that a critical SVF needs to be reached before the paste exits the barrel. Variations in extrudate LPR was evident when altering plunger rate and initial LPR. The differences in liquid content may be attributed to a longer residence time (for lower plunger rate) or higher powder permeability (for higher LPR). It does appear the critical SVF remains constant throughout extrusion (Fig. 5) regardless of pressure imposed on the bulk paste (Fig. 3). This indicates that phase separation due to filtration (mechanism (ii)) may not be the dominant mechanism, as variation in pressure would be expected to alter phase separation. Indeed, as the critical SVF is lower than the SVF of paste within the barrel, the particles must move apart to flow through the exit. This dilation would increase the voidage, reducing pore pressure in the exit region relative to the pore pressure upstream in the barrel. Therefore, liquid upstream would be drawn into fill the voids, indicating occurrence of mechanism (iii). Measurements of local (liquid) pore pressure would help to confirm which mechanism is dominant; however, these are difficult measurements to undertake at the scales observed in this study. Alternatively, mechanism (iii) has been modelled quantitatively by Patel [47] simulating the paste deformation using soil mechanics approaches, a similar approach is being considered going forward to establish the exact mechanism. Of course, both mechanisms (ii) and (iii) result in the paste within the barrel dewatering, until LPR_min_ is reached.

The apparent critical SVF required to flow, in parallel with the small variance in LPR_min_ throughout this investigation, highlights the importance of the packing fraction of CaP pastes. The packing fraction is a measure of how readily the powder packs to achieve the lowest voidage (highest SVF). Indeed, importance of SVF_max_ and SVF on the flowability of paste is well established [48]. At the same LPR, a powder with a high SVF_max_ will produce a paste that flows more readily than a powder with a low SVF_max_, due to a lower water demand. Bohner and Baroud [27], observed a relationship between injectability and the PL (related to SVF_max_). It was found the greater the difference between initial LPR and PL the greater the injectability. The SVF_max_ and PL are presented in Table 1.

Further work is required to determine the influence of packing ability on the flow properties of CaP pastes and cements, and indeed, the impact permeability and flow properties have on the phase separation mechanism. This will involve investigating whether characterisation and measuring methods used for cements and other biphasic pastes can be used with CaP pastes/cements. Knowledge and control of these properties will aid the design of injectable CaP pastes and CPC based systems that fully meet the clinical requirements of minimally invasive surgical application for bone augmentation.

## Conclusions

Phase separation was independent of needle geometry, excluding filtration in the needle as the dominant phase separation mechanism. Extrudate LPR remained constant throughout extrusion, but extrusion pressure did not; indicating pressure exerted by the plunger was not a determining factor of phase separation. These results are contrary to those expected for filtration within the syringe barrel, suggesting that local suction at the barrel exit is likely to be the dominant phase separation mechanism. Strategies to more definitively confirm this postulation include developing: (i) methods to monitor local pressure gradients before and after the barrel exit and (ii) computational models to analyse the biphasic mechanics of the system.

Formation of static zones during extrusion of CaP pastes was observed during extrusion with square ended dies. Tapering the barrel exit inhibited the formation of these features. As powder was not lost to the formation of static zones, when using tapered barrel exits, phase separation reduced, increasing injectability, revealing the use of tapered barrel exits as a successful improvement method.

This study has further highlighted that ‘injectability’ alone is an ineffective measure of extrusion for minimally invasive surgical applications. It was also highlighted powder packing ability and its impact on flow properties of CaP pastes require further attention to aid the design of injectable CaP pastes and CPC based systems that fully meet the clinical requirements of minimally invasive surgical application for bone augmentation.
